# Electronic cigarettes in standard smoking cessation treatment by tobacco counselors in Flanders: E-cigarette users show similar if not higher quit rates as those using commonly recommended smoking cessation aids

**DOI:** 10.1186/s12954-021-00475-7

**Published:** 2021-03-04

**Authors:** Karolien Adriaens, Eline Belmans, Dinska Van Gucht, Frank Baeyens

**Affiliations:** 1 Faculty of Psychology and Educational Sciences, KU Leuven – University of Leuven, Tiensestraat 102, 3000 Leuven, Belgium; 2 Thomas More University of Applied Sciences, Molenstraat 8, 2018 Antwerp, Belgium

**Keywords:** Longitudinal research, Tobacco harm reduction, Electronic cigarettes, NRT/medication/no aid vs. e-cigarettes

## Abstract

**Background:**

This interventional-cohort study tried to answer if people who smoke and choose an e-cigarette in the context of smoking cessation treatment by tobacco counselors in Flanders are achieving smoking abstinence and how they compare to clients who opt for commonly recommended (or no) aids (nicotine replacement therapy, smoking cessation medication).

**Methods:**

Participants were recruited by tobacco counselors. They followed smoking cessation treatment (in group) for 2 months. At several times during treatment and 7 months after quit date, participants were asked to fill out questionnaires and to perform eCO measurements.

**Results:**

One third of all participants (*n* = 244) achieved smoking abstinence 7 months after the quit date, with e-cigarette users having higher chances to be smoking abstinent at the final session compared to NRT users. Point prevalence abstinence rates across all follow-up measurements, however, as well as continuous and prolonged smoking abstinence, were similar in e-cigarette users and in clients having chosen a commonly recommended (or no) smoking cessation aid. No differences were obtained between smoking cessation aids with respect to product use and experiences.

**Conclusions:**

People who smoke and choose e-cigarettes in the context of smoking cessation treatment by tobacco counselors show similar if not higher smoking cessation rates compared to those choosing other evidence-based (or no) smoking cessation aids.

## Background

The effectiveness of electronic cigarettes (e-cigarettes) for smoking cessation has been studied using cross-sectional and longitudinal observational designs, as well as in Randomized Controlled Trials (RCTs). Firstly, cross-sectional data in convenience samples of current EU and US e-cigarette users indicate that around 80 to 90% of e-cigarette users report to have smoked in the past and that (regular) e-cigarette use is rare among people who never smoked [1–4]. In cross-sectional population data from the EU and UK, a large proportion (40–52%) of current daily e-cigarette users were found to be smoking abstinent [4–7]. Daily e-cigarette use is positively associated with smoking abstinence [8], and the increase in e-cigarette use is positively related to quit smoking attempts and to abstinence [8–10]. This positive relation has been confirmed in EU, UK and US data. Additionally, UK population data showed that e-cigarette users (and smoking cessation medication users) had higher odds to be smoking abstinent compared to those not using these aids [11]. Secondly, several well-conducted prospective and retrospective observational cohort studies from the US show that the likelihood of smoking abstinence is higher for those who smoke and self-select an e-cigarette in a quit attempt compared to those who do not [12–15]. Quit rates from such studies vary (both UK and US data), going from 20 to 52%; with the best results in regular and daily e-cigarette use, while using efficient e-cigarettes [16, 17]. Thirdly, the latest Cochrane review [18] from 2016, based on a limited number RCTs, concluded that although the number of RCTs is very low, e-cigarettes appear to be helpful in assisting smoking adults during their quit attempts. Since this review, two new RCTs have been conducted. First, Hajek and colleagues [19] investigated in 2019 the implementation of the e-cigarette in stop smoking services in the UK. Smoking participants signing up for smoking cessation treatment, were randomized to either using NRT or an e-cigarette during their quit attempt [19]. Compared to NRT users, e-cigarette users had higher smoking abstinence rates after one year (10% and 18%, respectively). Second, Walker and colleagues [20] reported in 2019 about their pragmatic, three-arm, parallel-group study wherein they randomized participants to using nicotine patches, nicotine patches in combination with nicotine-containing e-cigarettes, or nicotine patches in combination with nicotine-free e-cigarettes. Quit rates at 6 months were low (between 2 to 7%), but the overall conclusion was that combining nicotine patches with nicotine-containing e-cigarettes could modestly improve smoking cessation compared to using patches in combination with nicotine-free e-cigarettes [20]. In both RCTs [19, 20], second generation e-cigarettes (i.e. tank devices) were used with e-liquids containing nicotine concentrations between 10 and 18 mg/mL. The use of tank devices has been shown to be positively related with smoking cessation [21].

Overall, the majority of research is positive with respect to the effectiveness of e-cigarettes for smoking cessation. Nevertheless, the following question has rarely been addressed: *What is the effect on quit rates of adding the e-cigarette to the range of smoking cessation aids that people who smoke can choose from in standard smoking cessation treatment*? Individual and group-based (behavioral) smoking cessation programs (combined with the use of smoking cessation aids) appear to result in higher quit rates compared to self-help for smoking adults wanting to quit smoking, and there is a small additional benefit when cessation programs are combined with pharmacotherapy [22, 23]. However, the implementation of the e-cigarette in such programs is currently lacking (or negligible) in Flanders [24].

Smoking adults wanting to quit smoking, can do so with the support of a tobacco counselor in Flanders (i.e. health professional who completed a specific training to become a tobacco counselor) (www.tabakstop.be). Counseling can be individual or in group, and in combination with or without commonly recommended smoking cessation aids. In order to answer the aforementioned research question, we implemented the e-cigarette in the standard treatment provided by tobacco counselors. Previously (in 2016), we conducted a short-term pilot study (three month follow-up period, *n* = 53) which compared biochemically verified quit smoking rates between those who chose to use an e-cigarette and those who chose one of the commonly recommended (or no) smoking cessation aids [25]. Results showed that e-cigarette users, 3 months after quit date, had a higher chance to be completely smoking abstinent (RR = 1.69, 95% CI [1.03, 2.78], *p* < 0.05) compared to users of other (or no) smoking cessation aids. Although these results were promising, the pilot study had some limitations, such as the small sample size and a rather short follow-up period [25]. To overcome these limitations we conducted a similar trial using a larger sample size and a prolonged follow-up period. The main aim of the present study is to compare the quit rates of those choosing an e-cigarette to those choosing commonly recommended smoking cessation aids (or no aid) in the context of group-based smoking cessation treatment by a tobacco counselor.

## Methods

### Participants

Participants were recruited through five collaborating tobacco counselors from the Antwerp region (Flanders, Belgium) and were smoking adults who voluntarily followed smoking cessation group counseling. Treatment took place in several groups (group sizes: 5–17 people) starting in January, March and September 2017.

A total of 296 participants were reached, and 251 participants (85%) participated in at least the first or second follow-up assessment. Seven participants were excluded to prevent low numbers of participants in specific conditions (namely, e-cigarette plus medication, *n* = 2; medication plus NRT, *n* = 2; alternative aids, *n* = 3). Hence, the total sample included 244 participants (82% of 296), who were allocated to the different conditions (e-cigarette, NRT, e-cigarette + NRT, medication, no aid) depending on their choice of quit smoking aid(s) at FU1 (Follow-up 1; or FU2; see Additional file 1, Participants, for more detailed information) (*n*_e-cigarette_ = 70, *n*_NRT_ = 77, *n*_e-cigarette+NRT_ = 33, *n*_medication_ = 33, *n*_no aid_ = 31). Summarized, participants were allocated to the conditions depending on their own smoking cessation aid choice at FU1 or FU2, and no randomization took place.

### Procedure

This study was approved by the Societal and Social Ethics Committee of the University of Leuven (G-2016 03 515).

Smoking cessation groups followed the standard treatment protocol, which included eight sessions of about one hour and a half within a time window of 2 months. During each session, specific topics with respect to smoking cessation (e.g., explanation of smoking cessation aids, tips to prevent relapse) were covered by the counselors (see Additional file 1: Fig. 2).

At Intake (Session 1), participants were recruited and the informed consent was signed. Between Intake and FU1, participants received information concerning the available smoking cessation aids and could freely choose which aid they wanted to use. The quit date was planned between the third (Week 3) and fourth session (Week 4). Researchers were present at Intake, during the fifth (FU1) and seventh/eighth (FU2) session and asked participants to fill out a questionnaire and to perform an exhaled carbon monoxide (eCO) measurement. An additional follow-up (FU3) was organized on average 7 months after the quit date. At FU3, participants filled out a questionnaire, performed an eCO measurement, were debriefed about the study and presented with preliminary results. Finally, participants were compensated with one 50€ voucher from a large grocery store that was raffled among each 10 participants.

## Materials and outcome measures

### Physiological measures

The eCO measurements were performed using a piCO + Smokerlyzer® to biochemically verify smoking abstinence [26].

### Subjective effect questionnaires

The Intake questionnaire [2, 25, 27] assessed participants’ socio-demographics, smoking history and current smoking behavior, the Fagerström Test of Cigarette Dependence (FTCD) [28], the revised Minnesota Nicotine Withdrawal Scale (MNWS-R) [29], previous quit smoking attempts, the perceived harm of cigarettes, of commonly recommended smoking cessation aids and of e-cigarettes, the quit smoking motivation using the Reasons for Quitting questionnaire (RFQ) [30], the intended smoking cessation aid, and the provisional quit date. The first part of the follow-up questionnaires was identical for all participants and included questions similar to those used in the Intake questionnaire and the second part focused on the specific smoking cessation aid that participants were using. See Additional file 1, Materials and outcome measures, for all details.

The results presented here will mainly focus on the smoking behavior (i.e. quit rates, see Statistical analyses), product use and experiences at the end of treatment (i.e. FU2).

### Statistical analyses

See Additional file 1, Statistical analyses, for all details. Descriptive statistics of baseline characteristics were calculated (using Statistica, version 13) [31], and comparisons were made between conditions. We used one-way ANOVAs for continuous variables and Pearson’s chi square tests or Fisher’s exact tests (when expected *n* < 5 in more than 20% of the cells) for categorical variables. Alpha levels of 0.05 were used.

Our primary outcome measure included three smoking abstinence variables, as recommended by Hughes and colleagues [32], namely: Point prevalence abstinence, continuous smoking abstinence and prolonged smoking abstinence. Point prevalence abstinence (binary outcome: “quit” or “fail”) was determined for each participant at each FU based on self-reported quitting (0 cigarettes per day, CPD) verified by eCO measurement (eCO ≤ 7 ppm). Continuous smoking abstinence (i.e. being a quitter, or smoking abstinent, at each FU), and prolonged smoking abstinence (being a quitter, or smoking abstinent, from at least FU2 to the end of the study) for each participant were determined [32]. Due to high dropout rates, we conducted multiple imputation and 20 data sets were imputed [33]. Point prevalence abstinence rates were analyzed by mixed effect logistic regression models (using SAS, version 9.4) [34]. We fitted a mixed effects binomial logit-link model with an unstructured covariance matrix (i.e. time was specified as a categorical variable). A simple (including only time and condition, no covariates) and a complex model (covariates: having not achieved smoking abstinence with the currently used smoking cessation aid in the past, number of quit attempts in the past, longest quit period, FTCD, RFQ, interactions of these variables with condition, MNWS-R, CPD and eCO at Intake) were used to analyze point prevalence throughout time. Based on numerous other studies, the covariates were chosen based on their plausibility of being related to smoking cessation [35, 36]. Continuous and prolonged smoking abstinence were analyzed using logistic regression models. Furthermore, for each of the abstinence endpoints, sensitivity analyses using a pattern-mixture model approach were used to assess if results remained consistent if the assumption of missing ad random was violated. Additionally, post-hoc analyses were performed on point prevalence abstinence rates at FU2 and FU3 separately, using logistic regression models. Finally, Relative Risk ratio’s (RR) for point prevalence smoking abstinence at each FU, and continuous and prolonged smoking abstinence were calculated. Participants who were not present during a FU, were added to the “failures” (i.e. not achieved smoking abstinence). Corrections for multiple testing were also used when calculating the RR. Alpha levels were adjusted using the Bonferroni correction (alpha/*n*, where *n* is the number of comparisons used for each variable). The adjusted α-level was 0.005. Results of analyses before imputation are available in Additional file 2.

Secondary outcome measures included product use (e.g., brand of product, frequency of use) and overall product experiences (e.g., satisfaction with the aid, recommending the aid to others) at the end of treatment (i.e. FU2). Analyses included descriptive statistics similar to those described for baseline characteristics.

## Results

### Participants and baseline characteristics

Overall, participants were middle class smoking adults who were on average 52 years old (*SD* = 12.14) and a small majority was female (60%). All sociodemographic characteristics are displayed in Table 1 and no differences between conditions were observed for each of these characteristics, all *p*s > 0.11 (see Additional file 3: Table 1).

**Table 1:** Sociodemographic characteristics of the complete sample

Variable	*N*	*M* (*SD*) or %
**Demographic characteristics**		
Age (years)	243	52 (12.14)
Gender (men/women)	98/146	40.16/59.84
**Highest educational degree**		
None	11	4.51
Elementary school	7	2.87
High school	127	52.05
Non-academic bachelor	72	29.51
University	23	9.43
Other	2	0.82
Missing / did not wish to answer	2	0.82
**Occupation**		
Student	3	1.23
Part-time job	36	14.75
Full-time job	109	44.67
Housewife/-man	7	2.87
Job seeker	10	4.10
Retired	60	24.59
Invalidity	16	6.56
Missing / did not wish to answer	3	1.23
**Marital status**		
Single	33	13.52
Relationship, not living together	20	8.20
Relationship, living together	52	21.31
Married	96	39.34
Divorced	31	12.70
Widow(er)	9	3.69
Other	2	0.82
Missing / did not wish to answer	1	0.41
**Net income per month (in €)**		
< 1000	11	4.51
1000–1500	60	24.59
1500–2000	71	29.10
2000–2500	43	17.62
2500–3000	11	4.51
> 3000	5	2.05
Missing / did not wish to answer	43	17.62
**Nationality**		
Belgian	237	97.13
Other	4	1.64
Missing / did not wish to answer	3	1.23

On average, participants had started smoking at the age of 16 (*SD* = 3.63) and had been smoking regularly for around 31 years (*SD* = 13.56). Most had tried to quit smoking at least once (*n* = 202, 83%), with an average of four times (*SD* = 7.47) and with the longest quit period lasting on average around 21 months (*SD* = 37.34); no differences between conditions, all *p*s > 0.17. During the past quit smoking attempts, almost half of participants had ever tried to quit smoking using no aids (48%) or NRT (47%), 43% had ever tried smoking cessation medication, 19% had ever tried e-cigarettes, and 13% had ever tried to quit with counseling/support. The proportion of participants ever having used the currently chosen smoking cessation aid (or no aid), differed significantly between conditions, *χ*^*2*^(4) = 23.05, *p* < 0.001: Only 29% of current e-cigarette users had ever tried an e-cigarette in the past while 43% of current NRT users had ever tried NRT before, 61% of the no aid users also had used no aids before, 64% of medication users had used medication before, and 97% of the combination users had ever used e-cigarettes and/or NRT in the past.

At intake participants were smoking on average 16 CPD (*SD* = 7.70), had eCO levels of 22 ppm (*SD* = 12.79), were moderately cigarette dependent (*M*_FTCD_ = 4.73, *SD*_FTCD_ = 2.32), reported experiencing seldom to occasionally negative health effects of smoking (*M* = 1.58; *SD* = 0.72), reported little withdrawal symptoms (*M*_MNWS-R_ = 15.29; *SD*_MNWS-R_ = 10.69), and reported mainly intrinsic reasons to quit smoking (*M*_RFQ_ = 1.14, *SD*_RFQ_ = 0.73). No differences between conditions were observed concerning RFQ-scores, *F* < 1. Some between-condition differences did appear, however: No aid users smoked significantly less compared to all other conditions (all *p*s < 0.02), no aid users had lower eCO levels compared to medication users (*p* < 0.05) and combination users (*p* < 0.01), no aid users were less cigarette dependent compared to medication users (*p* < 0.01) and combination users (*p* < 0.001), NRT users were also less cigarette dependent compared to combination users (*p* < 0.01), and finally, combination users reported significantly more withdrawal symptoms compared to no aid users (*p* < 0.05).

With respect to dependence on smoking and nicotine, almost all participants reported feeling dependent on smoking and saw this as a problem (88%). A majority indicated that they would continue smoking if smoking was not harmful (66%). For both variables, differences between conditions were observed, both *p*s < 0.05: A larger proportion of no aid users (13%) felt not smoking dependent compared to e-cigarette users (0%), *χ*^*2*^(2) = 10.05, *p* < 0.01, with no differences between other conditions, all *p*s > 0.06; and larger proportions of e-cigarette users (76%) would continue smoking if not harmful compared to NRT users (57%), *χ*^*2*^(1) = 7.23, *p* < 0.01, and no aid users (55%), *χ*^*2*^(1) = 7.34, *p* < 0.01 (again no differences between other conditions, all *p*s > 0.07). Similarly, most participants felt nicotine dependent and saw this as a problem (80%), and would not continue using nicotine via a harmless way (66%), with again differences between conditions, both *p*s < 0.001: A larger proportion of no aid users (39%) reported not feeling nicotine dependent compared to e-cigarette (7%), NRT (5%), e-cigarette + NRT (0%), and medication (6%) users, all *p*s < 0.01. More e-cigarette (40%) and e-cigarette + NRT (42%) users expressed the intention to want to continue using nicotine via less harmful ways compared to NRT (22%), medication (15%), and no aid (10%) users, all *p*s < 0.05.

Harm perceptions were significantly different for cigarettes and each of the smoking cessation aids, *F*(3, 588) = 353.51, *p* < 0.001. The cigarette was perceived as more harmful compared to any other product, all *p*s < 0.001. In contrast, NRT was perceived as less harmful compared to any other product, all *p*s < 0.05. No differences between conditions were observed concerning harm perceptions of cigarettes, NRT and smoking cessation medication (all *p*s > 0.16). However, the harm of e-cigarettes was perceived differently between conditions, *F*(4, 200) = 5.67, *p* < 0.001, with e-cigarette users scoring the e-cigarette significantly less harmful compared to NRT users (*p* < 0.01) and to no aid users (*p* < 0.01).

## Effects of smoking cessation aid on abstinence outcomes

### Relative risks ratios

Point prevalence abstinence at each FU is presented in Table 2 and Relative Risk ratio’s (RR) for smoking abstinence are presented in Table 3. At FU1 and FU2, the RRs for smoking abstinence did not differ between conditions, all *p*s > 0.08 and all *p*s > 0.22, respectively. The only exception were medication users, who tended to have higher chances of smoking abstinence compared to NRT users at FU1, RR = 1.33 with 95% CI [1.00, 1.79], *p* = 0.05, and at FU2, RR = 1.47 with 95% CI [1.04, 2.07], *p* < 0.05. Overall, at FU3 no differences were found, all *p*s > 0.08, with the exception that e-cigarette users had higher chances to be smoking abstinent compared to NRT users, RR = 1.71 with 95% CI [1.04, 2.81], *p* < 0.05. Regarding continuous and prolonged smoking abstinence, no significantly different RRs were found between conditions, all *p*s > 0.13 and *p*s > 0.26, respectively. The only effect that was marginally significant, was that medication users tended to have higher chances to be smoking abstinent at all FUs (continuous) compared to NRT users, RR = 2.33 with 95% CI [0.96, 5.69], *p* = 0.06.

**Table 2:** Point prevalence abstinence at each FU

Condition	FU1			FU2			FU3			Continuous abstinence			Prolonged abstinence		
	Quit	Fail	Missing	Quit	Fail	Missing	Quit	Fail	Missing	Quit	Fail	Missing	Quit	Fail	Missing
E-cigarette	40 (57)	19 (27)	11 (16)	39 (56)	13 (19)	18 (26)	28 (40)	6 (9)	36 (51)	12 (17)	11 (16)	47 (67)	14 (20)	9 (13)	47 (67)
NRT	42 (55)	30 (39)	5 (7)	35 (46)	17 (22)	25 (33)	18 (23)	9 (12)	50 (65)	8 (10)	12 (16)	57 (74)	12 (16)	8 (10)	57 (74)
Medication	24 (73)	4 (12)	5 (15)	22 (67)	2 (6)	9 (27)	11 (33)	1 (3)	21 (64)	8 (24)	1 (3)	24 (73)	8 (24)	1 (3)	24 (73)
E-cigarette + NRT	17 (52)	13 (39)	3 (9)	18 (55)	10 (30)	5 (15)	11 (33)	5 (15)	17 (52)	7 (21)	7 (21)	19 (58)	8 (24)	6 (18)	19 (58)
No aid	19 (61)	7 (23)	5 (16)	17 (55)	3 (10)	11 (36)	10 (32)	4 (13)	17 (55)	6 (19)	4 (13)	21 (68)	6 (19)	4 (13)	21 (68)
All participants	142 (58)	73 (30)	29 (12)	131 (54)	45 (18)	68 (28)	78 (32)	25 (10)	141 (58)	41 (17)	35 (14)	168 (69)	48 (20)	28 (12)	168 (69)

“Quit” = smoking abstinent, “Fail” = not smoking abstinent, all *n*, % between () calculated based on the number of participants in the corresponding condition. *n*_E-cigarette_ = 70, *n*_NRT_ = 77, *n*_Medication_ = 33, *n*_E-cigarette+NRT_ = 33, *n*_No aid_ = 31, *n*_All participants_ = 244.

**Table 3:** Relative Risk ratio’s for smoking abstinence, all comparisons between conditions

Comparisons	FU1	FU2	FU3	Continuous abstinence	Prolonged abstinence
E-cigarette vs. NRT	1.05 [0.79–1.40] 0.75	1.23 [0.89–1.69] 0.22	1.71 [1.04–2.81] 0.03*	1.65 [0.72–3.80] 0.24	1.30 [0.65–2.62] 0.46
E-cigarette vs. Medication	0.79 [0.59–1.05] 0.10	0.84 [0.61–1.15] 0.27	1.20 [0.69–2.10] 0.52	0.71 [0.32–1.56] 0.39	0.83 [0.38–1.77] 0.62
E-cigarette vs. E-cigarette + NRT	1.11 [0.75–1.64] 0.60	1.02 [0.70–1.49] 0.91	1.20 [0.69–2.10] 0.52	0.81 [0.35–1.86] 0.62	0.83 [0.38–1.77] 0.62
E-cigarette vs. No aid	0.93 [0.66–1.32] 0.69	1.02 [0.69–1.49] 0.94	1.24 [0.69–2.23] 0.47	0.89 [0.37–2.14] 0.79	1.03 [0.44–2.44] 0.94
NRT vs. Medication	0.75 [0.56–1.00] 0.05	0.68 [0.48–0.96] 0.03*	0.70 [0.37–1.32] 0.27	0.43 [0.18–1.05] 0.06	0.64 [0.89–1.41] 0.26
NRT vs. E-cigarette + NRT	1.06 [0.72–1.56] 0.77	0.83 [0.56–1.24] 0.37	0.70 [0.37–1.32] 0.27	0.49 [0.19–1.24] 0.13	0.64 [0.89–1.41] 0.26
NRT vs. No aid	0.89 [0.63–1.26] 0.51	0.83 [0.55–1.24] 0.36	0.73 [0.38–1.39] 0.33	0.54 [0.20–1.42] 0.21	0.80 [0.33–1.93] 0.61
Medication vs. E-cigarette + NRT	1.41 [0.96–2.09] 0.08	1.22 [0.82–1.81] 0.32	1.00 [0.51–1.98] 1.00	1.14 [0.47–2.79] 0.77	1.00 [0.43–2.35] 1.00
Medication vs. No aid	1.19 [0.84–1.68] 0.34	1.22 [0.82–1.81] 0.34	1.03 [0.51–2.09] 0.93	1.25 [0.49–3.20] 0.64	1.25 [0.49–3.20] 0.64
No aid vs. E-cigarette + NRT	1.90 [0.77–1.84] 0.43	1.01 [0.64–1.57] 0.98	0.97 [0.48–1.95] 0.93	0.91 [0.35–2.42] 0.85	0.80 [0.31–2.04] 0.64
E-cigarette vs. All other conditions	0.98 [0.77–1.24] 0.83	1.05 [0.82–1.36] 0.68	1.39 [0.96–2.02] 0.08	1.03 [0.56–1.90] 0.93	1.02 [0.59–1.79] 0.34

All are RR, 95% CI and *p*-values. * *p* < 0.05. + *p* < 0.005, when standard alpha levels of 0.05 were adjusted familywise using the Bonferroni correction to 0.005. When absent at a FU, participants were included as “failure” (i.e. not achieved smoking abstinence).

Next, alpha levels were adjusted using Bonferroni correction (alpha/*n*, where *n* is the number of comparisons used for each variable) to correct for multiple testing. The adjusted α-level was 0.005. All previously reported effects between conditions then disappeared. At FU1, FU2 and FU3, the RRs for smoking abstinence did not differ between conditions, all *p*s > 0.05, > 0.03, and > 0.03, respectively. Overall, the same was found for continuous and prolonged smoking abstinence, all *p*s > 0.06, and > 0.26, respectively.

### Point prevalence abstinence at each FU

After multiple imputation and not controlling for covariates (see Table 4), quit rates remained stable over time (from FU1 to FU3), *F*(2, 156.20) = 0.69, *p* = 0.50, and no interaction effect between time and condition, *F*(8, 185.59) = 0.92, *p* = 0.50, was found. The effect of condition was only marginally significant, *F*(4, 2817.63) = 2.33, *p* = 0.05, with none of the comparisons between conditions being significant, all *p*s > 0.10. Sensitivity analyses revealed similar results: no effect of time, *F*(2, 216.18) = 0.80, *p* = 0.45; no effect of condition, *F*(4, 514.37) = 1.76, *p* = 0.14; and no interaction effect, *F*(8, 511.14) = 0.93, *p* = 0.49.

**Table 4:** Tests of fixed effects for primary outcome measures after imputation, and for sensitivity analyses

Effect	Analyses after imputation			Sensitivity analyses		
	*df*	*F*	*p*	*df*	*F*	*p*
**Point prevalence smoking abstinence**						
Time	2, 256.20	0.69	0.50	2, 216.18	0.80	0.45
Condition	4, 2817.63	2.33	0.05	4, 514.37	1.76	0.14
Condition*Time	8, 185.59	0.92	0.50	8, 511.14	0.93	0.49

Effect	Analyses after imputation			Sensitivity analyses		
	*df*	*t*-value	*p*	*df*	*t*-value	*p*
**Continuous smoking abstinence**						
Condition	273.33	0.15	0.88	165.97	−0.12	0.91
**Prolonged smoking abstinence**						
Condition	380.69	−0.39	0.70	297.89	−0.58	0.56

*p < 0.05, ** *p* < 0.01, *** *p* < 0.001

When controlling for specific covariates (see Additional file 4: Table 1), point prevalence rates remained stable over time, *F*(2, 248.39) = 1.15, *p* = 0.32, and no differences between conditions were observed, *F*(4, 187.35) = 0.99, *p* = 0.41. None of the covariates contributed significantly, all *p*s > 0.13; only baseline eCO tended to contribute, *F*(1, 153.28) = 4.07, *p* = 0.05, with having a lower eCO at intake resulting in being more likely to be smoking abstinent across time *t*(175.28) = -2.04, *p* < 0.05. These results remained stable when performing sensitivity analyses: no effect of time, *F*(2, 156.42) = 2.28, *p* = 0.11, no effect of condition, *F*(4, 1487.18) = 1.32, *p* = 0.26, no overall effects of covariates, all *p*s > 0.13, except for baseline eCO, *F*(1, 317.25) = 6.65, *p* < 0.05, with again participants with lower baseline eCO having higher overall abstinence rates, *t*(400.72) = -2.18, *p* < 0.05.

### Post-hoc analyses

In our pilot study [25], we observed a tendency that throughout treatment users of all aids were equal in achieving smoking cessation. At FU2 (end of treatment), however, differences were found between conditions. Therefore we expected to find an interaction between time and condition in previous mixed effect logistic regression models. Results of the current study, however, did not replicate these findings. Therefore post-hoc analyses for point prevalence abstinence at FU2 as well as at FU3 were performed.

No differences between conditions were observed for abstinence at FU2, *t*(185.02) = -0.88, *p* = 0.38, and FU3, *t*(169.39) = -0.39, *p* = 0.69, see Table 5. These results remained stable for both FU2 and FU3 when performing sensitivity analyses, *t*(242.41) = -0.65, *p* = 0.52, and, *t*(274.64) = -0.61, *p* = 0.54, respectively. The same analyses were carried out while controlling for several covariates, see Additional file 4 Table 2. Overall, for point prevalence abstinence at FU2 and FU3 separately, no differences were found between conditions, *t*(17256.00) = 0.18, *p* = 0.86, and, *t*(1.34^E6^) = 0.05, *p* = 0.96, respectively. None of the covariates were significant, all *p*s > 0.07. Finally, sensitivity analyses also did not show any differences between conditions with respect to point prevalence abstinence at both FU2, *t*(5664.60) = 0.01, *p* = 0.99, and FU3, *t*(2187.60) = 0.12, *p* = 0.91, and none of the covariates were significant, all *p*s > 0.10.

**Table 5:** Tests of fixed effects for point prevalence smoking abstinence at FU2 and FU3

	Analyses after imputation			Sensitivity analyses		
Effect	*df*	*t*-value	*p*	*df*	*t*-value	*p*
**Point prevalence smoking abstinence at FU2**						
Condition	185.02	−0.88	0.38	242.41	−0.65	0.52
**Point prevalence smoking abstinence at FU3**						
Condition	169.39	−0.39	0.69	274.64	−0.61	0.54

*p < 0.05, ** *p* < 0.01, *** *p* < 0.001

### Continuous and prolonged smoking abstinence

After imputation and not controlling for covariates (see Table 4), no differences between conditions were observed for continuous, *t*(273.33) = 0.15, *p* = 0.88, nor for prolonged smoking abstinence, *t*(380.69) = -0.39, *p* = 0.70. When controlling for several covariates (see Additional file 4: Table 1), the conditions did not differ for continuous smoking abstinence, *t*(157.54) = 0.51, *p* = 0.61, nor for prolonged abstinence, *t*(16,505.00) = 0.20, *p* = 0.84, and none of the covariates contributed to either abstinence rate, all *p*s > 0.08 and > 0.27, respectively. All aforementioned results remained stable after sensitivity analyses.

### Product use and experiences

Results of product use and experiences will only be presented for FU2, because at FU1 participants had only just started to use their smoking cessation aids, and at FU3 the dropout rates were high and not all participants were still using their aid.

At FU2, e-cigarette users were using up-to-date e-cigarettes, used e-liquids with nicotine levels of on average 5.86 mg/mL (*SD* = 3.82, min = 0.00 and max = 21.00), and consumed on average 17 mL e-liquid per week (*SD* = 12.34, min = 1.00 and max = 60.00). The majority of e-cigarette users were using their e-cigarette daily (84%, 57/68 participants), were taking less than 100 puffs per day (59%, 38/64 participants) or between 101 and 200 puffs per day (31%, 20/64 participants), reported wanting to reduce the nicotine levels to zero (73%, 48/66 participants), and reported wanting to quit using their e-cigarette completely (60%, 41/68 participants). Next, 37% (15/41 participants) of NRT users were using single slow-acting NRT (patches), 29% (12/41) used single fast-acting NRT (e.g., gum, inhaler), and the remaining 34% (14/41) used a combination of slow- and fast-acting NRT (e.g., patch plus gum). The nicotine concentration of these aids was on average 12.70 mg/sample (*SD* = 7.18, min = 2.00 and max = 26.00) and participants were using on average 2 samples per day (*SD* = 2.65). The majority of NRT users reported using their NRT daily (79%, 33/42 participants), planned to reduce the nicotine levels to zero (79%, 34/43 participants) and planned to completely quit using their NRT (70%, 30/43). Lastly, medication users were using their medication daily and were using varenicline (100%, 17/17 participants).

Results for the product experiences (experienced benefits, negative health effects, satisfaction and recommendation, and side effects for medication users) will only include data from exclusive e-cigarette, NRT and medication users, see Table 6 for all details. At FU2, the experienced benefits of the used smoking cessation aid did not differ between conditions, *F*(2, 85) = 1.27, *p* = 0.29. Participants reported experiencing occasionally to often benefits (e.g., could reduce/quit smoking, improved health) from their smoking cessation aid. Negative health effects (e.g., headache, sore throat) were experienced never to rarely, with no differences between conditions, *F*(2, 81) = 1.75, *p* = 0.18. Participants were also asked to report how satisfied they were with the smoking cessation aid they were using. Again, there were no differences between conditions, *F*(2, 86) = 1.86, *p* = 0.16. Participants in all three conditions reported being (highly) satisfied with their aid. Lastly, a similar pattern was observed concerning recommending the smoking cessation aid. No differences between conditions were found at FU2, *F*(2, 87) = 0.18, *p* = 0.84, and participants reported to strongly recommend the aid they were using to others. Finally, at FU2, medication users reported to experience never to rarely side effects of their medication (e.g., concentration difficulties, dizziness; *M* = 0.67, *SD* = 0.73).

**Table 6:** Experienced benefits, negative health effects, satisfaction of aid, and recommendation at FU2

Condition	Experienced benefits	Negative health effects	Satisfaction	Recommendation
E-cigarette	41 2.78 (0.11)	40 0.60 (0.09)	41 83.22 (3.86)	41 80.07 (3.96)
NRT	29 2.81 (0.13)	28 0.78 (0.11)	32 72.00 (4.37)	32 78.53 (4.48)
Medication	18 2.50 (0.16)	16 0.89 (0.15)	16 79.19 (6.18)	17 83.06 (6.15)

Conditions include exclusive users of specified smoking cessation aids. All are *n*, *M*s and SE between (). Experienced benefits and Negative health effects are average total scores going from 0 to 4. Satisfaction and Recommendation are VAS-scores with a 0 to 100 range.

Finally, based on the participants present at FU3, 79% (27/34) of e-cigarette users were still using their e-cigarette compared to 50% (8/16) of e-cigarette + NRT users, 22% (6/27) of NRT users, and 8% (1/12) of medication users.

## Discussion

### Summary main findings

One third of the total sample was biochemically verified smoking abstinent 7 months after quit date (FU3), with e-cigarette users (40%) having significantly higher chances to be smoking abstinent than NRT users (23%); in this analysis, missing data at FU3 were classified as “failures” (i.e. not achieved smoking abstinence). This finding is in line with the results of our pilot study [25]. However, when analyzing (the evolution of) point prevalence abstinence rates over time after multiple imputation of missing data (rather than working from the assumption that missing data equals “failure” or not achieved abstinence), no differences between conditions were found: Those who chose e-cigarettes achieved similar smoking cessation rates as those choosing other evidence-based smoking cessation aids. In addition, no clear results were found with respect to a range of plausibly contributing covariates, indicating that smoking cessation rates could not be predicted based on participants’ measured baseline characteristics.

Looking at continuous and prolonged smoking abstinence, 17% of the total sample was smoking abstinent at each FU (continuous), and 20% was smoking abstinent when including a grace period of two weeks (prolonged). Among e-cigarette users, these percentages were also 17% and 20%, respectively; again, no differences were obtained between conditions in any of the analyses. This is in contrast with the results of the recent RCT by Hajek and colleagues [19], where they did find a superior effect for smoking participants randomized to e-cigarettes in the context of stop-smoking treatment compared to those randomized to NRT. One-year continuous smoking abstinence rates, however, were similar to those achieved after 7 months in the current study. More specifically, in the study by Hajek and colleagues [19] 18% of e-cigarette users vs. 10% of NRT users were continuously smoking abstinent compared to 17% and 10%, respectively, in the current study. Apparently, when people who smoke have an equal willingness to use e-cigarettes or NRT, similar quit rates can be achieved when they are randomized to a particular aid compared to when they can self-select their smoking cessation aid. Also, again no superiority or inferiority could be demonstrated for those choosing e-cigarettes compared to other (or no) aid users with regard to continuous and prolonged smoking abstinence. Caponnetto and colleagues [37] have recently obtained similar results in a cohort study wherein they estimated the effect of smoking cessation aids in a real-life setting. Those who used an e-cigarette (not first-generation) had similar abstinence rates compared to NRT users. In addition, they replicated that using commonly recommended smoking cessation aids results in smoking abstinence when combined with counseling [37].

At FU2, irrespective of the smoking cessation aid used, participants on average reported little negative health effects, were satisfied by using the aid, would recommend their aid to others and reported a willingness to quit using these aids in the future. An important observation was that at FU2 more e-cigarette users (exclusive, or in combination with NRT) were still using their aid compared to those who had chosen another smoking cessation aid. This is similar to what Hajek and colleagues [19] had found. The fact that those who chose e-cigarettes continued to use their e-cigarette in the long-term can potentially explain the fact that, speaking in absolute terms, e-cigarette users had higher quit rates (and hence less relapse) 7 months after quit date. The use of NRT and smoking cessation medication is more restricted in duration of use. Guidelines typically include the recommendation to consume NRT or take the medication for only a specific amount of time, followed by quitting the use of these aids. It could be that, especially for long-term sustained smoking abstinence, this is not the most effective strategy. Continued, long-term use of NRT – as observed in many smoking people who completely switched to e-cigarettes – might be beneficial to avoid relapse. E-cigarettes, being consumer products rather than medicinal (prescription) products, are perceived by many users as a long-term alternative for smoking, which may contribute to less relapse.

### Contextualizing findings

The results obtained with regard to the absence of clear differences between conditions, should be interpreted in the context of the following considerations. Firstly, to stay close to the standard practices used in smoking cessation counseling, the current study was not designed as an RCT. Participants were free to choose a smoking cessation aid of their preference. The fact that participants self-selected for particular smoking cessation aids (or no aid), rather than being randomized to different conditions, has two implications for a correct interpretation of current observation of a lack of clear differences between the quit rates in different conditions. The first is that the lack of evidence for differences in the efficacy of the different aids, may either reflect a genuine equal efficacy, or alternatively, may follow from the fact that true differences are masked by residual confounding. Namely, we did include some covariates to control for potential confounding (that is, to minimize the influence on the smoking cessation outcome of variables correlated with the choice of a particular cessation aid and that by themselves may influence (facilitate/inhibit) the smoking cessation outcome), making it somewhat more likely that eventual differences (or lack of differences) in quit rates between different conditions could be attributed (at least in part) to the smoking cessation aid proper and not (only) to differences in confounding baseline characteristics. But, these covariates were mainly focused on and *limited to variables related to smoking/nicotine dependence and smoking intensity* (e.g., baseline FTCD-score, CPD, factors all known to affect smoking cessation outcomes). Therefore, it is still possible that some *other*, not directly smoking-related variables may have impacted the quit smoking rates; to the extent that those undetected but important variables may also have affected the choice of a particular cessation aid, *residual confounding* may have led to under- or over-estimation of the “real efficacy” of an aid. In turn, this may have exaggerated or masked differences in the true efficacy of different cessation aids. Such unmeasured confounding variable could be, for example, one’s belief in one’s own capacities to achieve smoking abstinence (“self-efficacy”). If high self-efficacy with regard to smoking cessation would predict higher a priori quit rates, and participants choosing “no aid” would be situated higher on self-efficacy than people choosing any particular aid, it would be wrong to infer from the absence of a difference in net quit rates between participants choosing “no aid” versus participants choosing a cessation aid, that the smoking cessation aid did not contribute to the quit rates (that is, those having chosen an aid might attain less smoking cessation without the aid than with the aid, and might show lower quit rates without the aid than was observed in the current study). A second consequence of the current study not being an RCT, is that the lack of difference between conditions with respect to the chosen cessation aid, should not be interpreted as indicating that the different aids would work equally well as observed here, nor that each of the aids would work equally well as any of the other aids, *if smoking adults were randomized to using these aids*. More likely, people who smoke will have self-selected for better-than-average quit rates with the particular cessation aid chosen (based on, for example, beliefs about and attitudes towards the product, personal goals with respect to future nicotine use, or a social environment supporting the use of that aid), such that randomizing people who smoke to cessation aids might result in lower quit rates than achieved with a self-selection strategy. For example, if smoking adults that chose e-cigarettes had been asked to use any other cessation aid, they might either have refused to use that alternative aid, or in case they would have accepted it, they might have had significantly lower chances to achieve smoking abstinence compared to when they actually could have chosen their preferred option.

Secondly, all participants in the current study were smoking adults who actively sought professional support to try to quit smoking. Among the smoking population this is a relative minority and it may well be the case that these people have other psychological/behavioral characteristics that are predictive of smoking cessation compared to those who try to quit by themselves. In other words, the current findings about the efficacy of different smoking cessation aids may not generalize to the majority of the smoking population who do not consider, or are actively refusing to get professional assistance when trying to quit smoking. Moreover, specifically related to vaping, e-cigarettes have also been related to “accidental quitting” [38]. That is, some people who smoke and who try out vaping or who are offered an e-cigarette by friends and have no intention to quit smoking, eventually quit smoking by switching to vaping [38].

Thirdly, the smoking cessation group sessions were provided by five different tobacco counselors. We did not have much control on how these counselors provided the information about the smoking cessation aids (including e-cigarettes) to the smoking participants. It could be that some of the counselors were more or less positive about, for example, medication compared to the other smoking cessation aids, which may have influenced not only the actual choice for medication, but also the impact on cessation rates with medication (the same is of course true for the potential impact of the different counselors’ preferences for any of the other cessation aids). It is also possible that specific group dynamics impacted the choice of smoking cessation aids and subsequently the chances of achieving smoking abstinence with any of the cessation aids. For example, if lots of participants in a particular group opted for, and were enthusiastic about, and ultimately were smoking abstinent with an e-cigarette, this may have enhanced the efficacy of e-cigarettes for any member haven chosen e-cigarettes in that particular group. To counter and to minimize the impact of potential different attitudes among the tobacco counselors regarding e-cigarettes, we organized an information session before data collection. During this session, we provided up-to-date scientific information regarding e-cigarettes and answered questions from the counselors. The main topics covered during the session included safety, efficacy, legislation, and practical issues regarding nicotine concentration, flavors and where to buy e-cigarettes.

Fourth and finally, a major weakness of the current study is that we had to deal with high numbers of incomplete follow-up data, especially at FU3. Nevertheless, multiple imputation was used to handle missing data. Results remained more or less robust, even after performing sensitivity analyses. Yet, results need to be seen in light of the used underlying models and their assumptions and limitations.

## Conclusions

This study provided evidence that in the context of smoking cessation treatment by tobacco counselors, a majority of smoking adults chose to use e-cigarettes to quit smoking. Those having chosen to use e-cigarettes achieved similar if not higher smoking abstinence rates as those opting for commonly recommended (or no) smoking cessation aids. Therefore, health professionals can feel confident that providing e-cigarettes as a smoking cessation aid in addition to the range of other already available evidence-based aids should not undermine a person’s chance of achieving abstinence from smoking.

## Supplementary Information

**Additional file 1**. Supplementary materials Methods-section. More detailed information (including additional tables) regarding the Methods.

**Additional file 2**. Results of analyses before imputation. Additional information regarding analyses done before multiple imputation.

**Additional file 3**. Supplementary materials Results-section. Large additional table regarding participants and baseline characteristics.

**Additional file 4**. Supplementary materials Results-section. Additional tables regarding the Results.

## Data Availability

The datasets used and/or analyzed during the current study are available from the corresponding author on reasonable request.

## Abbreviations

E-cigarettes: Electronic cigarettes
RCTs: Randomized Controlled Trials
NRT: Nicotine Replacement Therapy
RR: Relative Risk
eCO: Exhaled Carbon Monoxide
FU: Follow-up
FTCD: Fagerström Test for Cigarette Dependence
MNWS-R: Revised Minnesota Nicotine Withdrawal Scale
RFQ: Reasons for Quitting Questionnaire
CPD: Cigarettes Per Day
