# Supplementary material for: Electronic cigarettes in standard smoking cessation treatment by tobacco counselors in Flanders: E-cigarette users show similar if not higher quit rates as those using commonly recommended smoking cessation aids
Source: Harm Reduct J. 2021 Mar 4;18:28. doi: 10.1186/s12954-021-00475-7 (PMC7931336; doi:10.1186/s12954-021-00475-7)
Supplement: Supplementary file 1 — Additional file 1. Supplementary materials Methods-section. More detailed information (including additional tables) regarding the Methods. [file 12954_2021_475_MOESM1_ESM.doc]

**Additional File 1 – Supplementary materials Methods-section**

*Participants*

A total of 244 participants were included in the statistical analyses. Participants were only included when they completed the Intake questionnaire (+ Informed Consent) and at least the FU1 or FU2 questionnaire. The rationale behind this decision was that we needed to know which smoking cessation aid participants were actively using during their quit attempt, so that we could allocate them to the different conditions (e-cigarette, NRT, e-cigarette+NRT, medication, no aid). In general, participants were allocated to the conditions depending on their choice at FU1 (*n* = 215). For those not present at FU1, but present at FU2 (*n* = 29), allocation depended on their choice at FU2. Table 1 shows the distribution of attendance of the participants and Table 2 displays the distribution of the participants in conditions.

Overall, participants seldom changed their smoking cessation aid throughout the study, confirming our decision to allocate participants to the condition based on their choice at FU1 or FU2. The most important courses of participants’ smoking cessation aid choices were the following: continuing using the smoking cessation aid initially chosen, using no aid anymore or not being present at the subsequent follow up. See Table 3 and Figure 1 for all detailed information.

Table 1

*Attendance of participants for all sessions*

| **Attendance** | ***n* (% of sample)** |
| --- | --- |
| Intake + FU1^a^ | 50 (20.49) |
| Intake + FU2^b^ | 20 (8.20) |
| Intake + FU1 + FU2^a^ | 71 (29.10) |
| Intake + FU1 + FU3 | 18 (7.38) |
| Intake + FU2 + FU3^b^ | 9 (3.69) |
| Intake + FU1 + FU2 + FU3^a^ | 76 (31.15) |

*Legend*: ^a^ are mutual exclusive categories including at least FU1; ^b^ are mutual exclusive categories including at least FU2.

Table 2

*Distribution of participants in conditions*

| **Conditions** | ***n* (% of sample)** |
| --- | --- |
| E-cigarette | 70 (28.69) |
| NRT | 77 (31.56) |
| E-cigarette+NRT | 33 (13.52) |
| Medication | 33 (13.52) |
| No aid | 31 (12.70) |

Table 3

*Course of participants’ smoking cessation aid choices*

| **Course of aid choices between FUs** | **FU1 to FU2** | **FU2 to FU3** | **FU1 to FU3** |
| --- | --- | --- | --- |
| E-cigarette – E-cigarette | 38/59 (64.41) | 25/59 (42.37) | 26/59 (44.07) |
| E-cigarette – Missing | 19/59 (32.20) | 27/59 (45.76) | 30/59 (50.85) |
| E-cigarette – NRT |  | 1/59 (1.70) |  |
| E-cigarette – E-cigarette+NRT |  | 3/59 (5.09) |  |
| E-cigarette – No aid | 2/59 (3.39) | 2/59 (3.39) | 2/59 (3.39) |
| E-cigarette – Others |  | 1/59 (1.70) | 1/59 (1.70) |
| NRT – NRT | 35/72 (48.61) | 7/43 (16.28) | 48/72 (66.67) |
| NRT – missing | 25/72 (34.72) | 30/43 (69.77) | 14/72 (19.44) |
| NRT – E-cigarette+NRT | 1/72 (1.39) |  | 1/72 (1.39) |
| NRT – Medication | 1/72 (1.39) |  |  |
| NRT – No aid | 9/72 (12.50) | 6/43 (13.95) | 9/72 (12.50) |
| NRT – No idea | 1/72 (1.39) |  |  |
| E-cigarette+NRT – E-cigarette+NRT | 10/30 (33.33) | 5/14 (35.71) | 8/30 (26.67) |
| E-cigarette+NRT – Missing | 5/30 (16.67) | 7/14 (50.00) | 14/30 (46.67) |
| E-cigarette+NRT – E-cigarette | 8/30 (26.67) | 1/14 (7.14) | 4/30 (13.33) |
| E-cigarette+NRT – NRT |  |  | 1/30 (3.33) |
| E-cigarette+NRT – No aid | 1/30 (3.33) | 1/14 (7.14) | 3/30 (10.00) |
| E-cigarette+NRT – Others | 1/30 (3.33) |  |  |
| Medication – Medication | 15/28 (53.57) | 4/23 (17.39) | 4/28 (14.29) |
| Medication – Missing | 9/28 (32.14) | 14/23 (60.87) | 18/28 (64.29) |
| Medication – E-cigarette |  |  | 1/28 (3.57) |
| Medication – NRT |  | 1/23 (4.35) |  |
| Medication – No aid | 4/28 (14.29) | 3/23 (13.04) | 4/28 (14.29) |
| Medication – Others |  | 1/23 (4.35) | 1/28 (3.57) |
| No aid – No aid | 13/26 (50.00) | 15/34 (44.12) | 11/26 (42.31) |
| No aid – Missing | 11/26 (42.31) | 14/34 (41.18) | 14/26 (53.85) |
| No aid – E-cigarette | 2/26 (7.69) | 1/34 (2.94) |  |
| No aid – NRT |  | 4/34 (11.76) | 1/26 (38.46) |
| No idea – E-cigarette+NRT |  | 1/1 (100.00) |  |
| Others – E-cigarette |  | 1/1 (100.00) |  |
| Missing – E-cigarette | 11/29 (37.93) | 7/69 (10.15) | 4/29 (13.79) |
| Missing – NRT | 5/29 (17.24) | 3/69 (4.35) |  |
| Missing – E-cigarette+NRT | 3/29 (10.34) | 1/69 (1.45) | 1/29 (3.45) |
| Missing – Medication | 5/29 (17.24) | 1/69 (1.45) | 1/29 (3.45) |
| Missing – No aid | 5/29 (17.24) | 5/69 (7.25) | 3/29 (10.34) |
| Missing – Missing |  | 52/69 (75.36) | 20/29 (68.97) |

*Legend*: all are proportions with the numerator being the number of participants following the mentioned course of aid choices between FUs, and the denominator being the number of participants that had chosen the aid in the first mentioned FU. Between ( ) are %.


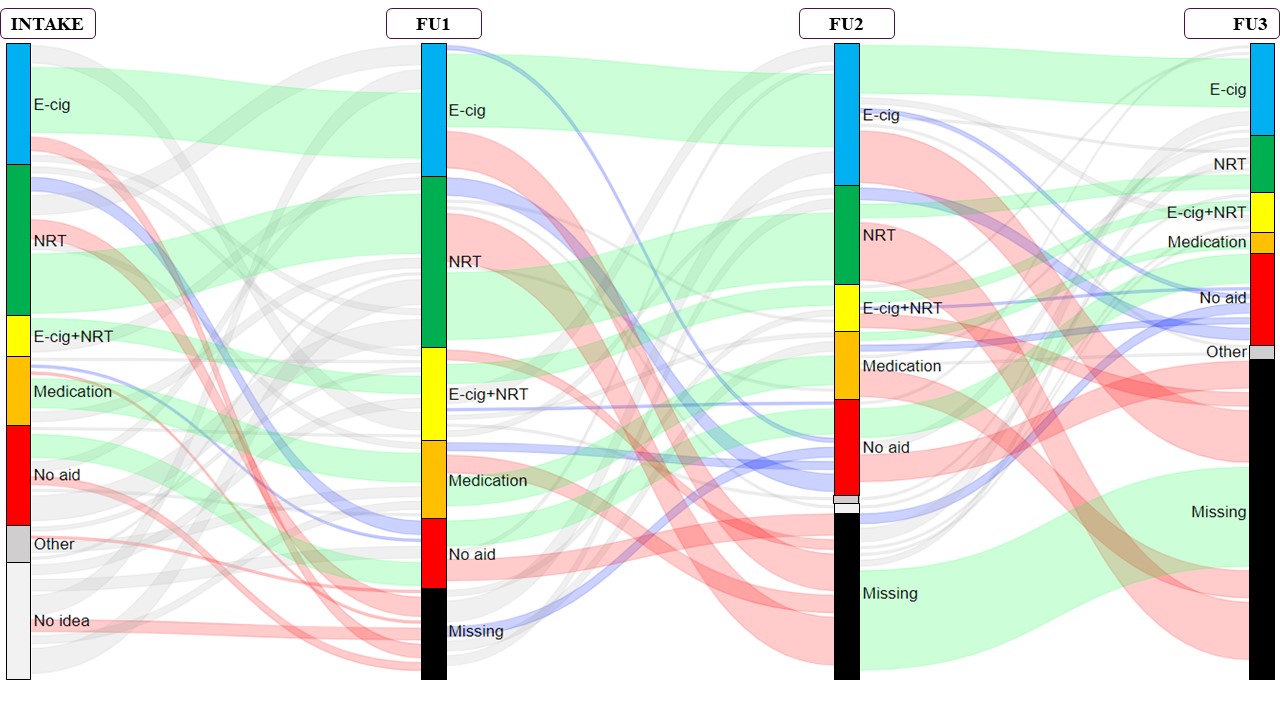


*Figure 1.* Course of participants’ smoking cessation aid choices

*Legend*: Green wavy lines are participants using the same aid from one FU to the following, red wavy lines are participants who were missing from one FU to the following, purple wavy lines are participants who go from using an aid to using no aid at the next FU, grey wavy lines include all other changes between FUs.


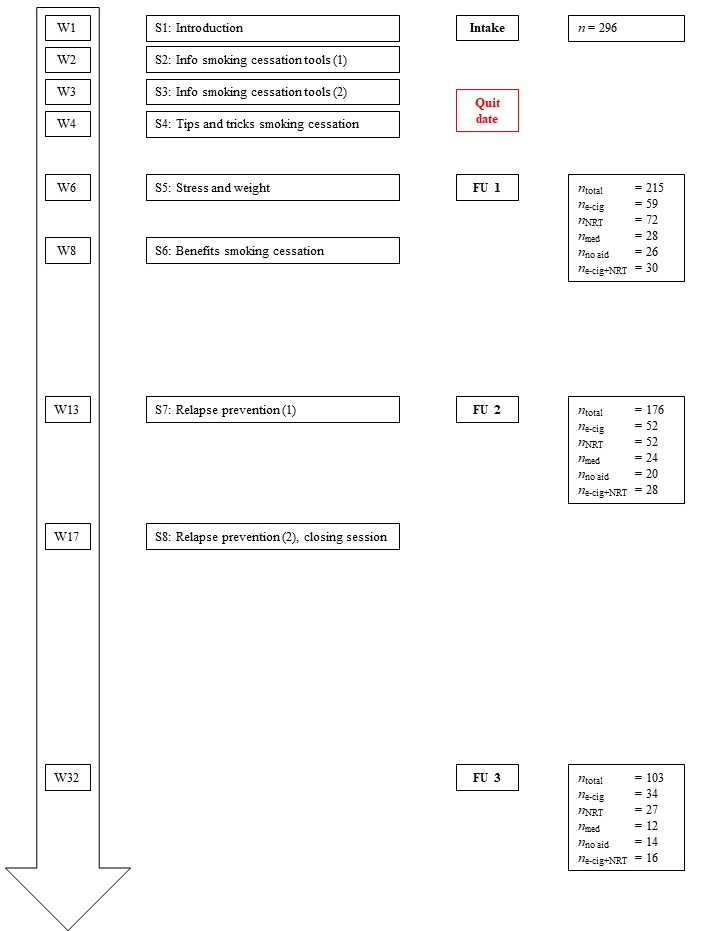


*Figure 2.* Procedure and participant flow.

*Materials and outcome measures – Subjective effect questionnaires*

- Intake questionnaire:
  - Socio-demographics: age, gender, education, occupation, marital status, income and nationality. All predefined categories with the option to provide additional information, except for age and nationality which were open questions. Participants could for each of these items indicate “*not willing to answer the question*”.
  - Smoking history: all open questions concerning age of first cigarette, age when regular smoking started, and number of years of regular smoking.
  - Current smoking behavior: open question for current number of cigarettes (smoked) per day (CPD); predefined categories for situations where smoking (when at home, alone, in company, drinking alcohol, drinking coffee, on the road, watching television, at a bar, at school/work, after a meal, immediately after getting up) and reasons why smoking (relaxation, to pass time, reducing stress, routine, nicotine craving, atmosphere, cigarette craving); 14 Likert scales from 0 “*Never*” to 4 “*Always*” for experienced negative health effects of smoking (headaches, sore throat, cough tendencies, dry mouth, dry throat, unpleasant throat sensation when inhaling, poor sense of taste, poor sense of smell, difficult breathing, increased palpitations, sleeping problems, bad condition, increased weight, pondering health); predefined categories concerning perceived addiction/dependence on smoking and on nicotine (feeling dependent on smoking: no, yes and this is not a problem, yes and this is a problem; continue smoking when cigarettes would not be harmful: no, yes; feeling dependent on nicotine: no, yes and this is not a problem, yes and this is a problem; continue using nicotine with a harmless method: no, yes). This set of questions was added with two standardized questionnaires, the Fagerström Test for Cigarette Dependence (FTCD)^1^ and the Revised Minnesota Nicotine Withdrawal Scale (MNWS-R)^2^. The FTCD contains six items to assess the level of dependence on cigarettes.^1^ The MNWS-R contains 15 items assessing withdrawal symptoms that are answered on a five-point scale from 0 “*None*” to 4 “*Sever*e”.^2^
  - Quit smoking attempts: open question concerning number of quit attempts and period of longest quit period in months. Predefined categories for smoking cessation aids used during quit attempts and longest quit period (e-cigarette with nicotine; NRT: gum, patches, lozenges, inhaler; medication; smoking cessation counseling; no aid).
  - Harm perceptions: Participants indicated, on Likert scales from 0 “*Not harmful at all*” to 10 “*Very harmful*”, the perceived harm of a tobacco cigarette, an e-cigarette, smoking cessation medication and NRT.
  - Quit smoking motivation: First, an open question was used in which participants described their overall reasons to quit smoking now. The Reasons for Quitting questionnaire assesses the intrinsic and extrinsic motivation for smoking cessation (RFQ).^3^ The RFQ contains 20 items that are answered on Likert scales from 0 “*Not at all true*” to 4 “*Extremely true*”. Four dimensions can be extracted and a level of intrinsic relative to extrinsic motivation can be calculated.
- Follow-up questionnaires:
  - The common part for all participants included questions that were similar to those used in the Intake questionnaire. The questions assessed the quit date, the current smoking cessation aid, current smoking status with a yes – no question (with only for current smoking participants an additional assessment of CPD and experienced negative health effects), perceived degree of addiction/dependence on smoking and on nicotine, harm perceptions, quit smoking motivation (including RFQ^3^), and withdrawal symptoms (MNWS-R)^2^.
  - Specific part smoking cessation aid – questions for all aids:
    - Still using aid or not: Yes – no question.
    - Brand: Open question.
    - Frequency of use: Predefined categories to assess how often participants were using their smoking cessation aid: “*Daily*”, “*Weekly, but not daily*”, and “*Several times per month*”.
    - Experienced benefits and negative health effects of using the aid: 14 Likert scales for experienced benefits going from 0 “*Totally disagree*” to 4 “*Completely agree*” (could decrease smoking, could quit smoking, craving for cigarette is reduced, smell/taste cigarette is less pleasant, less disturbing for other people, fitness and health are improved, fresher breath, improved taste, improved sense of smell, improved sleep quality, more often in a good mood, better breathing, improved appetite, less coughing) and 13 Likert scales for experienced negative health effects of smoking going from 0 ”*Never*” to 4 “*Always*” (headaches, sore throat, cough tendencies, dry mouth, dry throat, poor sense of taste, poor sense of smell, difficult breathing, increased palpitations, sleeping problems, bad condition, increased weight, pondering health).
    - Addiction/dependence on the aid: Three Likert scales going from 0 “*Totally disagree*” to 4 “*Completely agree*” (I feel dependent on the aid; I consider my dependence as problematic; I am dependent on the nicotine of the aid).
    - Degree of satisfaction and recommendation to others of the aid: Visual Analogue Scale (VAS) going from 0 “*Not at all satisfied*” to 100 “*Very satisfied*”, and from 0 “*Do not recommend at all*” to 100 “*Would definitely recommend*”, respectively.
  - Specific part smoking cessation aid – additional questions:
    - Type:
      - E-cigarette users: kind of battery and atomizer (open question).
      - NRT users: indicating which type of NRT (e.g., nicotine patches, nicotine gum, etc.).
    - Nicotine concentration: Open questions assessing nicotine concentration:
      - E-cigarette users: open question, nicotine concentration in e-liquid (mg/mL).
      - NRT users: open question, amount of nicotine per sample (mg/sample).
    - Only for e-cigarette users:
      - Puffs per day: predefined categories (≤ 100 puffs, 101-200 puffs, 201-300 puffs, ≥ 301 puffs).
      - Amount of e-liquid in mL: open question.
    - Only for NRT users:
      - Number of samples used per day: open question.
    - Future plans with e-cigarette/NRT and nicotine concentration. Both questions included predefined categories, more specific: going from “*Using more than now*” to “*Trying to completely quit*” and from “*Increasing nicotine concentration*” to “*Trying to reduce nicotine concentration, with in the end no more nicotine*”, respectively.
    - Side-effects: Only for medication, 13 Likert scales going from 0 ”*Never*” to 4 “*Always*” (concentration difficulties, tremor, sweating, anxiety, feelings of sadness, irritability, abnormal dreaming, drowsiness/fatigue, dizziness, nausea/vomiting/stomach ache, fever, rash/itch, increased appetite).

*Statistical analyses*

- The dependent variables include the following three smoking abstinence variables: Point prevalence abstinence, continuous smoking abstinence and prolonged smoking abstinence. Prolonged smoking abstinence was included to grant participants a grace period because not all participants immediately become smoking abstinent at the quit date.
- With respect to the multiple imputation procedure:
  - Missing data were imputed for 29 participants at FU1, 68 participants at FU2, and 141 participants at FU3. The number of missing data presented is the approximate number of missing data based on point prevalence abstinence.
- The complex model does not include the following interactions: condition X time, condition X MNWS-R, condition X CPD at baseline, and condition X eCO at intake. These were not included due to convergence problems by probably high multicollinearity between these variables.
- Additional analyses: In addition to the analyses presented in the main manuscript, we also carried out the same analyses on the sample before multiple imputation and thus including only complete cases. For point prevalence abstinence, when participants were not present during a specific FU, they were registered as missing for that measurement moment. With respect to continuous and prolonged abstinence, participants were registered as missing when they were not present at least during one of the FUs.

*References*

1. Fagerström K. Determinants of tobacco use and renaming the FTND to the Fagerström Test for Cigarette Dependence. *Nicotine Tob Res*. 2012;14(1):75-78. doi:10.1093/ntr/ntr137
2. Etter J-F, Ussher M, Hughes JR. A test of proposed new tobacco withdrawal symptoms. *Addiction.* 2012;108(1):50-59. doi:10.1111/j.1360-0443.2012.03981.x
3. Curry S, Wagner EH, Grothaus LC. Intrinsic and extrinsic motivation for smoking cessation. *J Consult Clin Psychol.* 1990;58(3):310-316. http://dx.doi.org/10.1037/0022-006X.58.3.310
