# Supplementary material for: Electronic cigarettes in standard smoking cessation treatment by tobacco counselors in Flanders: E-cigarette users show similar if not higher quit rates as those using commonly recommended smoking cessation aids
Source: Harm Reduct J. 2021 Mar 4;18:28. doi: 10.1186/s12954-021-00475-7 (PMC7931336; doi:10.1186/s12954-021-00475-7)
Supplement: Supplementary file 2 — Additional file 2. Results of analyses before imputation. Additional information regarding analyses done before multiple imputation. [file 12954_2021_475_MOESM2_ESM.doc]

**Additional File 2 – Results of analyses before imputation**

Here we present the analyses of the primary abstinence outcome measures done before multiple imputation.

*Point prevalence abstinence at each FU*

When using the mixed effect logistic regression models (i.e. including only data from complete cases) and not controlling for covariates, quit rates remained stable over time (from FU1 to FU3), *F*(2, 239) = 0.87, *p* = 0.42, but differences between conditions were observed, *F*(4, 239) = 3.49, *p* < 0.01, see Table 1. Across all FU moments, NRT users achieved lower smoking abstinence rates compared to medication users, *t*(239) = -3.37, *p* < 0.001, and compared to e-cigarette users, *t*(239) = -2.06, *p* < 0.05. E-cigarette+NRT users achieved lower rates compared to medication users, *t*(239) = -2.83, *p* < 0.01, and there was also a tendency that e-cigarette users achieved lower quit rates than medication users, *t*(239) = -1.96, *p* = 0.05. None of the other pairwise comparisons were significant, all *p*s > 0.09. No interaction effect was found between time and condition, *F*(8, 239) = 0.41, *p* = 0.91.

When controlling for specific covariates (including only complete cases; see Table 2 for all details) point prevalence remained stable over time, *F*(2, 162) = 0.58, *p* = 0.56, but differed between conditions, *F*(4, 162) = 4.36, *p* < 0.01. Regarding the differences between conditions, across all FU moments, medication users had higher point prevalence abstinence rates compared to NRT users, *t*(162) = 2.21, *p* < 0.05, e-cigarette users, *t*(162) = 2.03, *p* < 0.05, and e-cigarette+NRT users, *t*(162) = 2.04, *p* < 0.05. In addition, no aid users had higher overall abstinence rates compared to NRT users, *t*(162) = 2.43, *p* < 0.05. None of the other pairwise comparisons were significant, all *p*s > 0.08. With respect to the significant covariates, a significant contribution of FTCD-score, *F*(1, 162) = 9.15, *p* < 0.01, and interaction effect between condition and FTCD-score, *F*(4, 162) = 3.34, *p* < 0.05, was observed. More specifically, the effect of FTCD-score (i.e. the higher the FTCD-score, the lower the abstinence rate) on overall abstinence scores was different between no aid and e-cigarette users, *t*(162) = -3.16, *p* < 0.01. The association between higher FTCD-scores and lower abstinence rates was more pronounced for no aid users compared to e-cigarette users. There was a significant interaction between RFQ-score and condition, *F*(4, 162) = 4.90, *p* < 0.001. The association between RFQ-score and abstinence rates was different between no aid and e-cigarette users, *t*(162) = -2.23, *p* < 0.05. This association was negative for no aid users and reversed for e-cigarette users. Lastly, the interaction between the duration of the longest quit period and condition, *F*(4, 162) = 3.39, *p* < 0.05, was significant. The association between longest quit period and overall abstinence rates was different between no aid and e-cigarette users, *t*(162) = 2.65, *p* < 0.01. This association was positive for no aid users and reversed for e-cigarette users.

*Post-hoc analyses*

Using logistic regression models (including only data from participants who were present), and not controlling for covariates (see Table 1), no differences between conditions were found regarding point prevalence abstinence at FU2, Wald *χ^2^*(4) = 7.02, *p* = 0.14, and FU3, Wald *χ^2^*(4) = 3.90, *p* = 0.42. The same analyses were carried out while controlling for several covariates (see Table 2). Overall, for both point prevalence abstinence at FU2, Wald *χ^2^*(4) = 2.89, *p* = 0.58, and FU3, Wald *χ^2^*(4) = 1.93, *p* = 0.75, no differences were found between conditions, and none of the covariates were found to have a significant contribution, all *p*s > 0.20.

*Continuous and prolonged smoking abstinence*

Using logistic regression models, not controlling for covariates (see Table 1), no differences between conditions were found with respect to either continuous smoking abstinence, Wald *χ^2^*(4) = 4.96, *p* = 0.29, or prolonged smoking abstinence, Wald *χ^2^*(4) = 2.45, *p* = 0.65.

The same analyses were conducted when controlling for several covariates (see Table 2). Based on the logistic regression models, no differences were found between conditions regarding continuous abstinence rates, Wald *χ^2^*(4) = 1.50, *p* = 0.83, and none of the covariates contributed significantly to these rates, all *p*s > 0.18. A similar pattern was observed for prolonged abstinence, with no differences between conditions, Wald *χ^2^*(4) = 1.52, *p* = 0.82, and no contributing covariates, all *p*s > 0.17, the only exceptions being that both baseline CPD, Wald *χ^2^*(1) = 3.95, *p* = 0.05, and eCO level, Wald *χ^2^*(1) = 3.81, *p* = 0.05, were marginally significant. Participants with a higher baseline CPD, were more likely to be smoking abstinent at least at both FU2 and FU3 compared to participants with lower baseline CPD (estimate: 0.43). For baseline eCO the opposite was observed, the lower the baseline eCO, the higher the prolonged smoking abstinence rates were (estimate: -0.16).

Table 1

Tests of fixed effects for point prevalence smoking abstinence, point prevalence smoking abstinence at FU2 and FU3, continuous smoking abstinence, and prolonged smoking abstinence before imputation when not controlling for covariates

|  | **POINT PREVALENCE SMOKING ABSTINENCE** | | |
| --- | --- | --- | --- |
| **Effect** | ***df*** | ***F*** | ***p*** |
| Time | 2, 239 | 0.87 | 0.42 |
| Condition | 4, 236 | 3.49 | 0.009** |
| Condition*Time | 8, 239 | 0.41 | 0.91 |
|  |  |  |  |
|  | **POINT PREVALENCE SMOKING ABSTINENCE AT FU2** | | |
| **Effect** | ***df*** | ***Wald χ^2^*** | ***p*** |
| Condition | 4 | 7.02 | 0.14 |
|  |  |  |  |
|  | **POINT PREVALENCE SMOKING ABSTINENCE AT FU3** | | |
| **Effect** | ***df*** | ***Wald χ^2^*** | ***p*** |
| Condition | 4 | 3.90 | 0.42 |
|  |  |  |  |
|  | **CONTINUOUS SMOKING ABSTINENCE** | | |
| **Effect** | ***df*** | ***Wald χ^2^*** | ***p*** |
| Condition | 4 | 4.96 | 0.29 |
|  |  |  |  |
|  | **PROLONGED SMOKING ABSTINENCE** | | |
| **Effect** | ***df*** | ***Wald χ^2^*** | ***p*** |
| Condition | 4 | 2.45 | 0.65 |

*Legend*: * *p* < 0.05, ** *p* < 0.01, *** *p* < 0.001

Table 2

Tests of fixed effects for point prevalence smoking abstinence, point prevalence smoking abstinence at FU2, and FU3, continuous smoking abstinence, and prolonged smoking abstinence before imputation when controlling for covariates

|  | **POINT PREVALENCE SMOKING ABSTINENCE** | | |
| --- | --- | --- | --- |
| **Effect** | ***df*** | ***F*** | ***p*** |
| Time | 2, 162 | 0.58 | 0.56 |
| Condition | 4, 162 | 4.36 | 0.002** |
| eCO Intake | 1, 162 | 0.00 | 0.99 |
| CPD Intake | 1, 162 | 0.00 | 0.98 |
| #quit attempts | 1, 162 | 2.22 | 0.14 |
| Longest quit period | 1, 162 | 2.20 | 0.14 |
| Not achieved abstinence with current aid in the past | 1, 162 | 1.99 | 0.16 |
| FTCD | 1, 162 | 9.15 | 0.003** |
| RFQ | 1, 162 | 1.07 | 0.30 |
| MNWS-R | 1, 162 | 0.01 | 0.91 |
| Condition*#quit attempts | 4, 162 | 2.22 | 0.07 |
| Condition*Longest quit period | 4, 162 | 3.39 | 0.01* |
| Condition*Not achieved abstinence with current aid in the past | 4, 162 | 2.20 | 0.07 |
| Condition*FTCD | 4, 162 | 3.34 | 0.01* |
| Condition*RFQ | 4, 162 | 4.90 | 0.0009*** |
|  |  |  |  |
|  | **POINT PREVALENCE SMOKING ABSTINENCE AT FU2** | | |
| **Effect** | ***df*** | ***Wald χ^2^*** | ***p*** |
| Condition | 4 | 2.89 | 0.58 |
| eCO Intake | 1 | 0.09 | 0.77 |
| CPD Intake | 1 | 0.72 | 0.40 |
| #quit attempts | 1 | 0.06 | 0.81 |
| Longest quit period | 1 | 0.08 | 0.78 |
| Not achieved abstinence with current aid in the past | 1 | 0.12 | 0.73 |
| FTCD | 1 | 0.43 | 0.51 |
| RFQ | 1 | 0.00 | 0.97 |
| MNWS-R | 1 | 0.24 | 0.62 |
| Condition*#quit attempts | 4 | 5.58 | 0.23 |
| Condition*Longest quit period | 4 | 1.98 | 0.74 |
| Condition*Not achieved abstinence with current aid in the past | 4 | 1.91 | 0.75 |
| Condition*FTCD | 4 | 1.73 | 0.79 |
| Condition*RFQ | 4 | 6.00 | 0.20 |
|  |  |  |  |
|  | **POINT PREVALENCE SMOKING ABSTINENCE AT FU3** | | |
| **Effect** | ***df*** | ***Wald χ^2^*** | ***p*** |
| Condition | 4 | 1.93 | 0.75 |
| eCO Intake | 1 | 0.78 | 0.38 |
| CPD Intake | 1 | 0.80 | 0.37 |
| #quit attempts | 1 | 0.00 | 0.95 |
| Longest quit period | 1 | 0.00 | 0.99 |
| Not achieved abstinence with current aid in the past | 1 | 0.01 | 0.94 |
| FTCD | 1 | 0.01 | 0.93 |
| RFQ | 1 | 0.00 | 0.98 |
| MNWS-R | 1 | 0.96 | 0.33 |
| Condition*#quit attempts | 4 | 2.85 | 0.58 |
| Condition*Longest quit period | 4 | 2.37 | 0.67 |
| Condition*Not achieved abstinence with current aid in the past | 4 | 0.59 | 0.96 |
| Condition*FTCD | 4 | 1.91 | 0.75 |
| Condition*RFQ | 4 | 0.15 | 0.99 |
|  |  |  |  |
|  | **CONTINUOUS SMOKING ABSTINENCE** | | |
| **Effect** | ***df*** | ***Wald χ^2^*** | ***p*** |
| Condition | 4 | 1.50 | 0.83 |
| eCO Intake | 1 | 0.81 | 0.37 |
| CPD Intake | 1 | 1.82 | 0.18 |
| #quit attempts | 1 | 0.10 | 0.75 |
| Longest quit period | 1 | 0.00 | 0.97 |
| Not achieved abstinence with current aid in the past | 1 | 0.03 | 0.87 |
| FTCD | 1 | 0.04 | 0.83 |
| RFQ | 1 | 0.07 | 0.80 |
| MNWS-R | 1 | 0.94 | 0.33 |
| Condition*#quit attempts | 4 | 0.45 | 0.98 |
| Condition*Longest quit period | 4 | 1.26 | 0.87 |
| Condition*Not achieved abstinence with current aid in the past | 4 | 1.84 | 0.77 |
| Condition*FTCD | 4 | 1.53 | 0.82 |
| Condition*RFQ | 4 | 2.25 | 0.69 |
|  |  |  |  |
|  |  |  |  |
|  | **PROLONGED SMOKING ABSTINENCE** | | |
| **Effect** | ***df*** | ***Wald χ^2^*** | ***p*** |
| Condition | 4 | 1.52 | 0.82 |
| eCO Intake | 1 | 3.81 | 0.05 |
| CPD Intake | 1 | 3.95 | 0.05 |
| #quit attempts | 1 | 0.07 | 0.80 |
| Longest quit period | 1 | 0.00 | 0.99 |
| Not achieved abstinence with current aid in the past | 1 | 0.05 | 0.81 |
| FTCD | 1 | 0.00 | 0.96 |
| RFQ | 1 | 0.00 | 0.95 |
| MNWS-R | 1 | 1.91 | 0.17 |
| Condition*#quit attempts | 4 | 1.25 | 0.87 |
| Condition*Longest quit period | 4 | 0.27 | 0.99 |
| Condition*Not achieved abstinence with current aid in the past | 4 | 4.69 | 0.32 |
| Condition*FTCD | 4 | 1.79 | 0.78 |
| Condition*RFQ | 4 | 0.39 | 0.98 |

*Legend*: * *p* < 0.05, ** *p* < 0.01, *** *p* < 0.001.

*Discussion*

When analyzing the evolution of point prevalence abstinence rates (not controlling for covariates) over time, and limiting the analysis to complete cases, medication users achieved higher quit rates across FU measurements compared to NRT, e-cigarette, and e-cigarette+NRT users. Part of this observation is in line with findings from population data that using medication during a quit attempt results in higher quit rates than (single) NRT use.^1,2^

Based on the data of the participants who were present at each follow-up moment (i.e. complete cases), several covariates were predictive for point prevalence, continuous, and prolonged smoking abstinence. After data imputation these effects disappeared, indicating that interpreting the impact of these covariates should be done with caution. After imputation, the only covariate that was predictive for point prevalence smoking abstinence was baseline eCO: The lower the eCO level at intake, the higher the overall quit rates.

*References*

1. Jackson SE, Kotz D, West R, Brown J. Moderators of real-world effectiveness of smoking cessation aids: A population study. *Addiction*. 2019;114(9):1627-1638. doi:10.1111/add.14656
2. Kotz D, Brown J, West R. ‘Real-world’ effectiveness of smoking cessation treatments: A population study. *Addiction*. 2013;109(3):491-499. doi:10.1111/add.12429
