# Supplementary material for: Electronic cigarettes in standard smoking cessation treatment by tobacco counselors in Flanders: E-cigarette users show similar if not higher quit rates as those using commonly recommended smoking cessation aids
Source: Harm Reduct J. 2021 Mar 4;18:28. doi: 10.1186/s12954-021-00475-7 (PMC7931336; doi:10.1186/s12954-021-00475-7)
Supplement: Supplementary file 3 — Additional file 3. Supplementary materials Results-section. Large additional table regarding participants and baseline characteristics. [file 12954_2021_475_MOESM3_ESM.doc]

**Additional File 3 – Supplementary materials Results: Participants and baseline characteristics**

Table 1

Sociodemographic and smoking-related baseline characteristics of the complete sample and each condition separately

| **Variable** | **E-cigarette**  ***n* = 70** | | **NRT**  ***n* = 77** | | **Medication**  ***n* = 33** | | **E-cigarette+NRT**  ***n* = 33** | | **No aid**  ***n* = 31** | | **All participants**  ***n* = 244** | |
| --- | --- | --- | --- | --- | --- | --- | --- | --- | --- | --- | --- | --- |
|  | ***n*** | ***M* (*SD*) or %** | ***n*** | ***M* (*SD*) or %** | ***n*** | ***M* (*SD*) or %** | ***n*** | ***M* (*SD*) or %** | ***n*** | ***M* (*SD*) or %** | ***n*** | ***M* (*SD*) or %** |
| **Demographic characteristics** |  |  |  |  |  |  |  |  |  |  |  |  |
| Age (years)  Gender (men/women) | 70  25 / 45 | 50.94 (11.72)  35.71 / 64.29 | 77  33 / 44 | 54.17 (12.52)  42.86 / 57.14 | 33  16 / 17 | 48.73 (10.45)  48.48 / 51.52 | 33  11 / 22 | 51.76 (12.40)  33.33 / 66.67 | 30  13 / 18 | 48.37 (12.76)  41.94 / 5806 | 243  98 / 146 | 52 (12.14)  40.16 / 59.84 |
| **Highest educational degree** |  |  |  |  |  |  |  |  |  |  |  |  |
| None  Elementary school  High school  Non-academic bachelor  University  Other  Missing / did not wished to answer | 2  1  41  17  8  1  0 | 2.86  1.43  58.57  24.29  11.43  1.43  0.00 | 6  1  34  26  8  1  1 | 7.79  1.30  44.16  33.77  10.39  1.30  1.30 | 1  3  18  10  1  0  0 | 3.03  9.09  54.55  30.30  3.03  0.00  0.00 | 1  2  13  12  4  0  1 | 3.03  6.06  39.39  36.36  12.12  0.00  3.03 | 1  0  21  7  2  0  0 | 3.23  0.00  67.74  22.58  6.45  0.00  0.00 | 11  7  127  72  23  2  2 | 4.51  2.87  52.05  29.51  9.43  0.82  0.82 |
| **Occupation** |  |  |  |  |  |  |  |  |  |  |  |  |
| Student  Part-time job  Full-time job  Housewife/-man  Job seeker  Retired  Invalidity  Missing / did not wished to answer | 1  14  32  2  2  2  16  1 | 1.43  20.00  45.71  2.86  2.86  2.86  22.86  1.43 | 1  7  29  1  4  9  25  1 | 1.30  9.09  37.66  1.30  5.19  11.69  32.47  1.30 | 0  6  19  0  1  3  3  1 | 0.00  18.18  57.58  0.00  3.03  9.09  9.09  3.03 | 0  4  14  3  0  2  10  0 | 0.00  12.12  42.42  9.09  0.00  6.06  30.30  0.00 | 1  5  15  1  3  0  6  0 | 3.23  16.13  48.39  3.23  9.68  0.00  19.35  0.00 | 3  36  109  7  10  60  16  3 | 1.23  14.75  44.67  2.87  4.10  24.59  6.56  1.23 |
| **Marital status** |  |  |  |  |  |  |  |  |  |  |  |  |
| Single  Relationship, not living together  Relationship, living together  Married  Divorced  Widow(er)  Other  Missing / did not wished to answer | 11  6  17  24  8  3  1  0 | 15.71  8.57  24.29  34.29  11.43  4.29  1.43  0.00 | 12  7  15  32  6  4  0  1 | 15.58  9.09  19.48  41.56  7.79  5.19  0.00  1.30 | 4  0  11  13  5  0  0  0 | 12.12  0.00  33.33  39.39  15.15  0.00  0.00  0.00 | 3  3  5  12  7  2  1  0 | 9.09  9.09  15.15  36.36  21.21  6.06  3.03  0.00 | 3  4  4  15  5  0  0  0 | 9.68  12.90  12.90  48.39  16.13  0.00  0.00  0.00 | 33  20  52  96  31  9  2  1 | 13.52  8.20  21.31  39.34  12.70  3.69  0.82  0.41 |
| **Net income per month (in €)** |  |  |  |  |  |  |  |  |  |  |  |  |
| <1000  1000-1500  1500-2000  2000-2500  2500-3000  >3000  Missing / did not wished to answer | 3  21  22  11  2  1  10 | 4.29  30.00  31.43  15.71  2.86  1.43  14.29 | 3  17  20  12  4  1  20 | 3.90  22.08  25.97  15.58  5.19  1.30  25.98 | 0  10  11  7  3  1  1 | 0.00  30.30  33.33  21.21  9.09  3.03  3.03 | 4  4  10  6  2  0  7 | 12.12  12.12  30.30  18.18  6.06  0.00  21.21 | 1  8  8  7  0  2  5 | 3.23  25.81  25.81  22.58  0.00  6.45  16.13 | 11  60  71  43  11  5  43 | 4.51  24.59  29.10  17.62  4.51  2.05  17.62 |
|  |  |  |  |  |  |  |  |  |  |  |  |  |
| **Nationality** |  |  |  |  |  |  |  |  |  |  |  |  |
| Belgian  Other  Missing / did not wished to answer | 69  1  0 | 98.57  1.43  0.00 | 72  2  3 | 93.51  2.60  3.90 | 32  1  0 | 96.97  3.03  0.00 | 33  0  0 | 100.00  0.00  0.00 | 31  0  0 | 100.00  0.00  0.00 | 237  4  3 | 97.13  1.64  1.23 |
| **Smoking history** |  |  |  |  |  |  |  |  |  |  |  |  |
| Age of first cigarette  Years smoking regularly  Tried to quit smoking in the past (Y/N)  Number of quit attempts  Duration longest quit attempt (months)  Smoking cessation aids ever used  E-cigarette *******  NRT *****  Medication *******  Counseling  No aid ****** | 70  70  52 / 18  48  48  52  20  22  20  7  28 | 15.64 (2.91)  31.03 (12.80)  74.29 / 25.71  2.92 (2.33)  22.46 (37.75)  38.46  42.31  42.31  13.46  53.85 | 74  75  65 / 12  59  64  65  4  33  22  10  32 | 15.54 (3.35)  32.03 (14.62)  84.42 / 15.58  3.07 (2.55)  21.29 (37.54)  6.15  50.77  33.85  15.39  49.23 | 33  33  27 / 6  26  27  27  1  13  21  3  7 | 15.36 (1.78)  30.64 (11.12)  81.82 / 18.18  2.62 (2.00)  19.13 (37.48)  3.70  48.15  77.78  11.11  25.93 | 33  33  30 / 3  27  29  29  12  20  15  5  11 | 15.18 (1.89)  32.80 (13.84)  90.91 / 9.09  3.00 (2.06)  14.44 (26.91)  41.38  68.97  51.72  17.24  37.93 | 30  31  28 / 3  25  27  26  1  6  6  1  19 | 17.03 (7.07)  26.61 (14.51)  90.32 / 9.68  6.76 (19.48)  25.10 (46.45)  3.85  23.08  23.08  3.85  73.08 | 240  242  202 / 42  185  195  199  38  94  86  26  97 | 15.68 (3.63)  30.96 (13.56)  82.79 / 17.21  3.45 (7.47)  20.79 (37.34)  19.10  47.24  43.22  13.07  48.74 |
| Ever use of current aid | 20/70 | 28.57 | 33/77 | 42.86 | 21/33 | 63.64 | 32/33 | 96.97 | 19/31 | 61.29 | 125 / 244 | 51.23 |
| **Smoking dependence at Intake** |  |  |  |  |  |  |  |  |  |  |  |  |
| Cigarettes smoked per day ******  Carbon monoxide ******  Experienced negative health effects  FTCD *******  RFQ  MNWS-R *****  Dependent on smoking *****  No  Yes and it is no problem  Yes and it is a problem  Missing  Continuing smoking when not harmful *****  No  Yes  Missing  Dependent on nicotine *******  No  Yes and it is no problem  Yes and it is a problem  Missing  Continuing nicotine use *******  No  Yes  Missing | 70  68  69  70  66  69  0  5  62  3  11  53  6  5  6  55  4  38  28  4 | 16.08 (8.17)  21.10 (12.14)  1.67 (0.66)  4.67 (2.12)  1.14 (0.69)  14.46 (10.48)  0.00  7.14  88.57  4.29  15.71  75.71  8.57  7.14  8.57  78.57  5.71  54.29  40.00  5.71 | 75  77  76  77  71  73  4  3  66  4  27  44  6  4  4  65  4  55  17  5 | 16.43 (6.79)  20.35 (11.30)  1.64 (0.74)  4.42 (2.29)  1.19 (0.80)  16.14 (10.72)  5.19  3.90  85.71  5.19  35.06  57.14  7.79  5.19  5.19  84.42  5.19  71.43  22.08  6.49 | 33  33  33  33  32  33  0  3  30  0  10  22  1  2  3  28  0  26  5  2 | 19.18 (6.65)  25.33 (12.26)  1.59 (0.70)  5.46 (2.31)  1.01 (0.65)  14.09 (9.61)  0.00  9.09  90.91  0.00  30.30  66.67  3.03  6.06  9.09  84.85  0.00  78.79  15.15  6.06 | 33  33  32  33  30  32  0  0  32  1  7  25  1  0  1  31  1  15  14  4 | 18.79 (8.83)  27.12 (13.86)  1.44 (0.71)  5.91 (1.79)  1.14 (0.83)  19.81 (11.61)  0.00  0.00  96.97  3.03  21.21  75.76  3.03  0.00  3.03  93.94  3.03  45.45  42.42  12.12 | 31  31  31  31  30  28  4  1  25  1  13  17  1  12  1  15  3  27  3  1 | 11.42 (6.18)  16.16 (14.56)  1.34 (0.79)  3.58 (2.67)  1.18 (0.59)  11.39 (9.92)  12.90  3.23  80.65  3.23  41.94  54.84  3.23  38.71  3.23  48.39  9.68  87.10  9.68  3.23 | 242  242  241  244  229  235  8  12  215  9  68  161  15  23  15  194  12  161  67  16 | 16.38 (7.70)  21.63 (12.79)  1.58 (0.72)  4.73 (2.32)  1.14 (0.73)  15.29 (10.69)  3.28  4.92  88.11  3.69  27.87  65.98  6.15  9.43  6.15  79.51  4.92  65.98  27.46  6.56 |
| **Harm perception** |  |  |  |  |  |  |  |  |  |  |  |  |
| Cigarette  E-cigarette *******  Medication  NRT | 68  64  60  63 | 9.12 (1.31)  3.77 (2.15)  4.50 (2.35)  4.05 (2.53) | 73  55  63  65 | 9.23 (1.26)  5.40 (2.30)  4.46 (2.50)  3.35 (2.45) | 33  30  32  32 | 9.00 (1.39)  4.70 (2.44)  3.91 (2.53)  4.19 (2.32) | 33  30  30  30 | 8.91 (1.68)  4.27 (2.13)  4.43 (2.86)  3.43 (2.46) | 31  26  24  24 | 9.00 (1.51)  5.85 (2.82)  4.92 (2.75)  4.63 (2.89) | 238  205  209  214 | 9.09 (1.38)  4.68 (2.43)  4.44 (2.54)  3.84 (2.52) |

*Note*: ***** *p* < 0.05, ****** *p* < 0.01, ******* *p* < 0.001
