# Supplementary material for: Electronic cigarettes in standard smoking cessation treatment by tobacco counselors in Flanders: E-cigarette users show similar if not higher quit rates as those using commonly recommended smoking cessation aids
Source: Harm Reduct J. 2021 Mar 4;18:28. doi: 10.1186/s12954-021-00475-7 (PMC7931336; doi:10.1186/s12954-021-00475-7)
Supplement: Supplementary file 4 — Additional file 4. Supplementary materials Results-section. Additional tables regarding the Results. [file 12954_2021_475_MOESM4_ESM.doc]

**Additional File 4 – Supplementary materials Results-section**

Table 1

Tests of fixed effects for point prevalence smoking abstinence, continuous smoking abstinence, and prolonged smoking abstinence after imputation and for sensitivity analyses when controlling for covariates

|  | **POINT PREVALENCE SMOKING ABSTINENCE** | | | | | | |
| --- | --- | --- | --- | --- | --- | --- | --- |
|  | **Analyses after imputation** | | | | **Sensitivity analyses** | | |
| **Effect** | ***df*** | ***F*** | ***p*** | | ***df*** | ***F*** | ***p*** |
| Time | 2, 248.39 | 1.15 | 0.32 | | 2, 156.42 | 2.28 | 0.11 |
| Condition | 4, 187.35 | 0.99 | 0.41 | | 4, 1487.18 | 1.32 | 0.26 |
| eCO Intake | 1, 153.28 | 4.07 | 0.05 | | 1, 317.25 | 6.65 | 0.03* |
| CPD Intake | 1, 1897.48 | 0.99 | 0.32 | | 1, 1233.69 | 0.81 | 0.37 |
| #quit attempts | 1, 147.91 | 1.69 | 0.20 | | 1, 158.72 | 1.47 | 0.23 |
| Longest quit period | 1, 219.73 | 0.88 | 0.35 | | 1, 130.01 | 0.69 | 0.41 |
| Not achieved abstinence with current aid in the past | 1, 164.76 | 0.64 | 0.43 | | 1, 175.78 | 0.59 | 0.44 |
| FTCD | 1, 436.40 | 0.56 | 0.46 | | 1, 616.60 | 0.61 | 0.43 |
| RFQ | 1, 156.88 | 2.30 | 0.13 | | 1, 93.85 | 1.99 | 0.16 |
| MNWS-R | 1, 544.70 | 0.52 | 0.47 | | 1, 323.12 | 0.34 | 0.56 |
| Condition*#quit attempts | 4, 107.06 | 0.69 | 0.60 | | 4, 106.39 | 0.68 | 0.61 |
| Condition*Longest quit period | 4, 117.26 | 1.51 | 0.20 | | 4, 146.00 | 1.81 | 0.13 |
| Condition*Not achieved abstinence with current aid in the past | 4, 343.50 | 0.73 | 0.57 | | 4, 218.59 | 0.85 | 0.50 |
| Condition*FTCD | 4, 212.30 | 1.44 | 0.22 | | 4, 695.76 | 1.79 | 0.13 |
| Condition*RFQ | 4, 273.24 | 0.80 | 0.53 | | 4, 242.38 | 0.82 | 0.52 |
|  |  |  |  | |  |  |  |
|  | **CONTINUOUS SMOKING ABSTINENCE** | | | | | | |
|  | **Analyses after imputation** | | | | **Sensitivity analyses** | | |
| **Effect** | ***df*** | ***t*-value** | | ***p*** | ***df*** | ***t*-value** | ***p*** |
| Condition | 157.54 | 0.51 | | 0.61 | 13281 | 0.14 | 0.89 |
| eCO Intake | 84.40 | -1.80 | | 0.08 | 133.00 | -1.80 | 0.07 |
| CPD Intake | 297.05 | -0.40 | | 0.69 | 322.37 | -0.41 | 0.68 |
| #quit attempts | 101.21 | 0.37 | | 0.71 | 558.49 | 0.40 | 0.69 |
| Longest quit period | 76.87 | 0.13 | | 0.89 | 133.95 | 0.03 | 0.98 |
| Not achieved abstinence with current aid in the past | 90.37 | -0.21 | | 0.83 | 685.20 | 0.00 | 0.99 |
| FTCD | 84.52 | -0.21 | | 0.83 | 563.93 | -0.17 | 0.87 |
| RFQ | 77.26 | 0.38 | | 0.71 | 1674.40 | 0.21 | 0.83 |
| MNWS-R | 153.80 | -0.53 | | 0.60 | 143.23 | -0.70 | 0.49 |
| Condition*#quit attempts | 90.19 | 0.24 | | 0.81 | 609.37 | 0.27 | 0.79 |
| Condition*Longest quit period | 74.33 | 0.16 | | 0.87 | 210.47 | 0.15 | 0.88 |
| Condition*Not achieved abstinence with current aid in the past | 80.13 | -0.22 | | 0.83 | 731.97 | -0.06 | 0.96 |
| Condition*FTCD | 87.80 | -0.28 | | 0.78 | 576.25 | -0.19 | 0.85 |
| Condition*RFQ | 74.20 | 0.17 | | 0.87 | 1905.30 | 0.10 | 0.92 |
|  |  |  | |  |  |  |  |
|  | **PROLONGED SMOKING ABSTINENCE** | | | | | | |
|  | **Analyses after imputation** | | | | **Sensitivity analyses** | | |
| **Effect** | ***df*** | ***t*-value** | | ***p*** | ***df*** | ***t*-value** | ***p*** |
| Condition | 16505 | 0.20 | | 0.84 | 515.69 | 0.17 | 0.87 |
| eCO Intake | 55.45 | -1.00 | | 0.32 | 232.52 | -1.17 | 0.25 |
| CPD Intake | 254.43 | -0.42 | | 0.68 | 306.00 | -0.25 | 0.80 |
| #quit attempts | 73.51 | 1.12 | | 0.27 | 226.87 | 0.22 | 0.82 |
| Longest quit period | 42.49 | -0.02 | | 0.99 | 252.86 | -0.11 | 0.91 |
| Not achieved abstinence with current aid in the past | 500859.00 | -0.09 | | 0.93 | 5276.1 | -0.02 | 0.99 |
| FTCD | 69.54 | -0.48 | | 0.63 | 275.57 | -0.15 | 0.88 |
| RFQ | 67.12 | 1.06 | | 0.29 | 333.83 | 0.26 | 0.79 |
| MNWS-R | 79.25 | -0.47 | | 0.64 | 100.18 | -0.57 | 0.57 |
| Condition*#quit attempts | 64.13 | 0.57 | | 0.57 | 231.89 | 0.16 | 0.87 |
| Condition*Longest quit period | 36.53 | -0.03 | | 0.97 | 255.01 | -0.11 | 0.91 |
| Condition*Not achieved abstinence with current aid in the past | 2784.10 | -0.07 | | 0.95 | 357.51 | 0.07 | 0.95 |
| Condition*FTCD | 55.64 | -0.79 | | 0.43 | 260.03 | -0.16 | 0.87 |
| Condition*RFQ | 46.89 | -0.06 | | 0.95 | 362.15 | 0.09 | 0.93 |

*Legend*: * *p* < 0.05, ** *p* < 0.01, *** *p* < 0.001.

Table 2

Tests of fixed effects for point prevalence smoking abstinence at FU2 and FU3 after imputation, and for sensitivity analyses when controlling for covariates

|  | **POINT PREVALENCE SMOKING ABSTINENCE AT FU2** | | | | | |
| --- | --- | --- | --- | --- | --- | --- |
|  | **Analyses after imputation** | | | **Sensitivity analyses** | | |
| **Effect** | ***df*** | ***t*-value** | ***p*** | ***df*** | ***t*-value** | ***p*** |
| Condition | 17256.00 | 0.18 | 0.86 | 5664.60 | 0.01 | 0.99 |
| eCO Intake | 281.50 | -1.25 | 0.21 | 250.94 | -1.64 | 0.10 |
| CPD Intake | 136.22 | -0.86 | 0.39 | 442.04 | -0.54 | 0.59 |
| #quit attempts | 851.86 | 0.01 | 0.99 | 130.97 | 0.19 | 0.85 |
| Longest quit period | 62201.00 | 0.03 | 0.97 | 260515.00 | 0.02 | 0.99 |
| Not achieved abstinence with current aid in the past | 709461.00 | -0.05 | 0.96 | 1.39^E6^ | -0.04 | 0.97 |
| FTCD | 279.82 | -0.51 | 0.61 | 1273.5 | -0.11 | 0.91 |
| RFQ | 306.4 | 0.29 | 0.77 | 1852.30 | 0.15 | 0.88 |
| MNWS-R | 199.84 | 0.76 | 0.45 | 295.42 | 1.21 | 0.23 |
| Condition*#quit attempts | 778.69 | 0.15 | 0.88 | 147.56 | 0.19 | 0.85 |
| Condition*Longest quit period | 740.53 | 0.16 | 0.87 | 418691.00 | 0.02 | 0.98 |
| Condition*Not achieved abstinence with current aid in the past | 11946.00 | -0.06 | 0.96 | 1.48^E7^ | 0.00 | 0.99 |
| Condition*FTCD | 90.05 | -0.73 | 0.47 | 1209.40 | -0.11 | 0.91 |
| Condition*RFQ | 171.75 | -0.12 | 0.91 | 1967.9 | 0.06 | 0.96 |
|  | **POINT PREVALENCE SMOKING ABSTINENCE AT FU3** | | | | | |
|  | **Analyses after imputation** | | | **Sensitivity analyses** | | |
| **Effect** | ***df*** | ***t*-value** | ***p*** | ***df*** | ***t*-value** | ***p*** |
| Condition | 1.34^E6^ | 0.05 | 0.96 | 2187.60 | 0.12 | 0.91 |
| eCO Intake | 93.34 | -0.37 | 0.71 | 181.78 | -0.01 | 0.99 |
| CPD Intake | 175.58 | -0.69 | 0.49 | 276.68 | -0.91 | 0.36 |
| #quit attempts | 101.27 | 1.06 | 0.29 | 94.46 | 0.41 | 0.69 |
| Longest quit period | 129.84 | -0.04 | 0.97 | 65.95 | -0.26 | 0.80 |
| Not achieved abstinence with current aid in the past | 4.63^E6^ | -0.07 | 0.94 | 373551.00 | -0.09 | 0.92 |
| FTCD | 96.21 | 0.48 | 0.63 | 677.95 | 0.02 | 0.98 |
| RFQ | 95.62 | 0.04 | 0.97 | 251.25 | 0.02 | 0.98 |
| MNWS-R | 141.77 | -1.83 | 0.07 | 102.78 | -1.41 | 0.16 |
| Condition*#quit attempts | 59.39 | -0.18 | 0.86 | 88.79 | 0.18 | 0.85 |
| Condition*Longest quit period | 88.91 | -0.51 | 0.61 | 66.73 | -0.26 | 0.80 |
| Condition*Not achieved abstinence with current aid in the past | 1.56^E6^ | 0.00 | 0.99 | 6171.50 | -0.06 | 0.95 |
| Condition*FTCD | 70.41 | -1.07 | 0.29 | 417.92 | -0.43 | 0.67 |
| Condition*RFQ | 124.52 | -0.65 | 0.51 | 314.82 | -0.10 | 0.92 |

*Legend*: * *p* < 0.05, ** *p* < 0.01, *** *p* < 0.001.
